# Stereotactic Arrhythmia Radioablation (STAR) of Ventricular Tachycardia: A Treatment Planning Study

**DOI:** 10.7759/cureus.694

**Published:** 2016-07-15

**Authors:** Lei Wang, Benjamin Fahimian, Scott G Soltys, Paul Zei, Anthony Lo, Edward A Gardner, Patrick J Maguire, Billy W Loo Jr.

**Affiliations:** 1 Department of Radiation Oncology, Stanford University School of Medicine; 2 Cardiology, Stanford University School of Medicine; 3 R&D, CyberHeart Inc.; 4 CyberHeart Inc.; 5 Stanford Cancer Institute, Stanford University School of Medicine

**Keywords:** stereotactic arrhythmia radioablation, stereotactic ablative radiotherapy, star, sabr, treatment planning, cyberknife, cardiac radiosurgery, sbrt

## Abstract

**Purpose:**

The first stereotactic arrhythmia radioablation (STAR) of ventricular tachycardia (VT) was delivered at Stanford on a robotic radiosurgery system (CyberKnife® G4) in 2012. The results warranted further investigation of this treatment. Here we compare dosimetrically three possible treatment delivery platforms for STAR.

**Methods:**

The anatomy and target volume of the first treated patient were used for this study. A dose of 25 Gy in one fraction was prescribed to the planning target volume (PTV). Treatment plans were created on three treatment platforms: CyberKnife® G4 system with Iris collimator (Multiplan, V. 4.6)(Plan #1), CyberKnife® M6 system with InCise 2^TM^ multileaf collimator (Multiplan V. 5.3)(Plan #2) and Varian TrueBeam^TM^ STx with HD 120^TM^ MLC and 10MV flattening filter free (FFF) beam (Eclipse planning system, V.11) (Plan #3 coplanar and #4 noncoplanar VMAT plans). The four plans were compared by prescription isodose line, plan conformity index, dose gradient, as well as dose to the nearby critical structures. To assess the delivery efficiency, planned monitor units (MU) and estimated treatment time were evaluated.

**Results:**

Plans #1-4 delivered 25 Gy to the PTV to the 75.0%, 83.0%, 84.3%, and 84.9% isodose lines and with conformity indices of 1.19, 1.16, 1.05, and 1.05, respectively. The dose gradients for plans #1-4 were 3.62, 3.42, 3.93, and 3.73 with the CyberKnife® MLC plan (Plan #2) the best, and the TrueBeam^TM^ STx co-planar plan (Plan #3) the worst. The dose to nearby critical structures (lung, stomach, bowel, and esophagus) were all well within tolerance. The MUs for plans #1-4 were 27671, 16522, 6275, and 6004 for an estimated total-treatment-time/beam-delivery-time of 99/69, 65/35, 37/7, and 56/6 minutes, respectively, under the assumption of 30 minutes pretreatment setup time. For VMAT gated delivery, a 40% duty cycle, 2400MU/minute dose rate, and an extra 10 minutes per extra arc were assumed.

**Conclusion:**

Clinically acceptable plans were created with all three platforms. Plans with MLC were considerably more efficient in MU. CyberKnife® M6 with InCise 2^TM^ collimator provided the most conformal plan (steepest dose drop-off) with significantly reduced MU and treatment time. VMAT plans were most efficient in MU and delivery time. Fluoroscopic image guidance removes the need for additional fiducial marker placement; however, benefits may be moderated by worse dose gradient and more operator-dependent motion management by gated delivery.

## Introduction

Ventricular arrhythmias (VA) are common complications of structural heart disease that cause significant morbidity and mortality [1]. The most common form of VA is monomorphic ventricular tachycardia (VT). Current available options for limiting the incidence of VT and VA include implantable cardioverter-defibrillators (ICDs) [2-4], antiarrhythmic medications [5-6], and catheter ablation [7]. Although effective, each intervention may entail significant side effects/complications.

Stereotactic ablative radiotherapy (SABR) is highly focused radiation therapy that targets well-demarcated, limited-volume malignant or benign tumors with high accuracy and precision using image guidance [8-11]. stereotactic arrhythmia radioablation (STAR) that applies SABR to treating arrhythmias was recently investigated. Pre-clinical studies have demonstrated electrophysiologic conduction blockade and histologic fibrosis after SABR to the targeted cardiac conduction pathway, which provided proof of principle for its potential for treating arrhythmias [12-13]. The first STAR of VT was treated at Stanford on a robotic radiosurgery system (CyberKnife®, Accuray, Sunnyvale, CA) in 2012 [14]. The follow-up showed no definite acute or late complications, and a seven-month reduction in VT on a stable antiarrhythmic regimen suggested a possible transient benefit of STAR; however, further investigation is needed.

The first STAR patient was treated on a CyberKnife® G4 system--a robotic radiosurgery system with an X-band linear accelerator mounted on a robotic arm. The radiation was delivered with a dynamic respiratory tracking system (Synchrony® Respiratory Tracking, Accuray, Sunnyvale, CA) to compensate for heart movement from respiratory motion. The treatment plan was created and optimized using Multiplan V.4.6 with Iris variable aperture collimator. The plan contained 175 non-isocentric beams with estimated delivery time of 69 minutes excluding setup time. A temporary pacing wire (Oscor, Inc., Miami Lakes, FL) was fluoroscopically placed in the RV apex as an imaging fiducial marker for tracking. The magnitude of the remaining cardiac motion was determined by fluoroscopy of the fiducial marker during transient breath holds, and the final target volume was expanded to encompass this residual motion.

Recent technological advances may enable faster and more conformal STAR treatment. The recently released CyberKnife® M6™ system is now equipped with multi-leaf collimator (InCise 2™) [15-16], which is reported to be more efficient in treating larger targets with fewer monitor units and less treatment time [17-19]. Linac-based SABR with gated VMAT delivery and a very high dose rate (flattening filter free) has also been implemented in the past several years. In this work, we perform a STAR treatment plan comparison study between three available treatment platforms.

## Materials and methods

### System description

**Figure 1:**
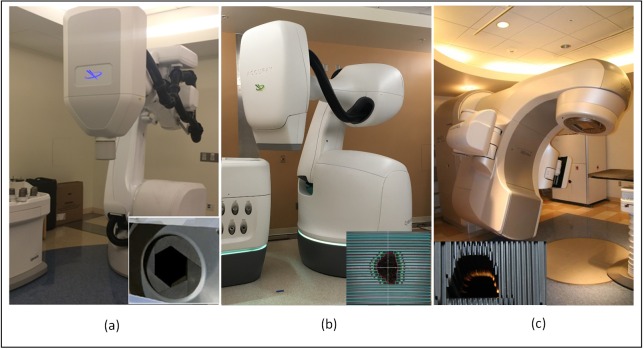
Three Available SABR Platforms (a) CyberKnife® G4^TM^ system with Iris^TM^ variable aperture collimator, (b) CyberKnife® M6^TM^ system with InCise 2^TM^ MLC, (c) Varian TrueBeam^TM ^STx with HD 120^TM^ MLC.

Three available SABR platforms were compared in this study as shown in Figure 1. The first was the CyberKnife® G4^TM^ system with an Iris variable aperture collimator which was used for our first STAR treatment. This system uses a 6 MV flattening filter free (FFF) photon beam with a dose rate of 1000 cGy/minute at 80 cm SAD. The Iris variable aperture is composed of two hexagonal leaf banks stacking on top of one another with 15-degree rotation. It can form twelve quasi-circular collimator sizes to mimic the conventional fixed cones. The application of Iris collimator is reported to reduce treatment time significantly compared to the conventional cones while creating comparable or better plans [20]. The second system was a CyberKnife® M6^TM^ system with an InCise 2^TM^ MLC [15]. The M6^TM^ system has a redesigned robot and a new geometric room layout. With the robot aligned with the couch, the new design opens more delivery space laterally and provides a more symmetrical delivery node distribution. More importantly, the M6^TM^ system is equipped with an optional InCise^TM^ MLC. The InCise 2^TM^ (second version of InCise MLC) has two leaf banks with 26 MLC leaves in each leaf bank. Each leaf has a width of 3.85mm at source-to-axis distance (SAD) of 80 cm with a maximum field size of 11.5 cm by 10.0 cm. The addition of MLC to CyberKnife® enables the system to produce plans with reduced MUs and reduced treatment time. The M6 system linear accelerator has similar beam characteristics as the G4 system. Both M6 and G4 systems use the Synchrony® model-based real-time tracking for respiratory motion. The third system was the Varian TrueBeam^TM^ STx with HD 120^TM^ MLC consisting of 2.5 mm leaf width (at 100 cm SAD) at the central 8 cm, and 5 mm width leaves in the periphery. This system has 6 MV and 10 MV FFF beams with dose rates of 1400 cGy/minute and 2400 cGy/minute, respectively. The 10 MV photon has higher dose rate and better penetration which is more appropriate for a deeply seated target which justifies the use of 10 MV photons in this study. The respiratory motion is typically compensated with gating technology with the aid of KV on-board imaging, cone-beam CT, and fluoroscopic imaging.   

### Target delineation

The anatomy of the first treated patient was used for this study. This was a 71-year-old man who developed sustained VT refractory to other treatment options. As he was not a candidate for catheter ablation, he consented to STAR treatment protocol as the first patient treatment in 2012 under IRB approval and FDA expanded use (i.e., compassionate use) exemption. In order to determine the ablation volume, a study including ECG during VT, cardiac-gated CT, and cardiac PET-CT were performed before the treatment. The circumferential VT substrate was contoured by the electrophysiologist using CardioPlan software (CyberHeart, Portola Valley, CA) [14]. The target and the images in CardioPlan were then exported to the CyberKnife® treatment planning system (MultiPlan 4.6, Accuray, Sunnyvale, CA), and fused with the treatment planning CT. The final target volume was refined by the radiation oncologists in collaboration with electrophysiology and nuclear medicine. Nearby normal organs including the lung, bowel, esophagus, and stomach were also delineated. The heart movement was measured to be about 1 cm with fluoroscopic imaging on the inserted ICD and tracking lead during the simulation. An internal target volume (ITV) was created to include a 5 mm margin to compensate for heart movement, and the planning target volume (PTV) was a modification of the ITV by the radiation oncologist considering geometry delivery feasibility (including the concave region to PTV for easier dosimetric delivery). In this study, the simulation CT scan of this patient and the original target and critical structure volumes were transferred to MultiPlan V.5.3 and Eclipse V.11 for replanning.

### Treatment planning 

A dose of 25 Gy in one fraction was prescribed to the PTV. Four treatment plans were created. Plan #1 was the original plan used for patient treatment that was created on the CyberKnife® G4 system with Iris collimator (Multiplan, V.4.6.0). Plan #2 was created with CyberKnife® M6 system with InCise 2^TM^ multi-leaf collimator (Multiplan V.5.3). Plans #3 and #4 were created on Varian TrueBeam^TM ^STx with HD 120^TM^ MLC and 10 MV FFF beam (Eclipse planning system, V.11). Plan #3 was a VMAT plan with one full arc in the axial plane. Plan #4 was a VMAT plan with one axial full arc and two anterior partial arcs (120 degrees anterior) with a 10-degree couch kick on each side to introduce superior and inferior noncoplanar angles. All four plans were optimized to be conformal to the PTV and meet dose constraints on the nearby critical structures following published dose constraints (AAPM Task Group 101). Plans were optimized to have a prescription isodose line between 75% to 85% (corresponding to dose heterogeneity of 133% and 118%). A beam- or segment-reduction technique was applied to both Cyberknife plans after the optimization. The system settings for the four plans are shown in Table 1. The plans were compared with respect to prescription isodose lines, plan conformity index, dose gradient, as well as dose to the nearby critical structures. The conformity index was defined as the volume of 100% of the prescription dose to the volume of PTV. The dose gradient was defined as the ratio of the volume of 50% of the prescription dose to the volume of the 100% prescription dose (i.e., the 12.5 Gy isodose volume over the 25 Gy isodose volume). To assess the delivery efficiency, plan monitor unit (MU) and estimated treatment time were also compared. The treatment time with the CyberKnife® was estimated in the planning software plus 30 minutes setup time. Treatment time on TrueBeam was an estimation based on our experience on gated treatments. The beam delivery time was calculated with the assumption of a dose rate of 2400 MU/minute at 40% duty cycle plus 30 minutes setup time ahead of treatment. The VMAT plan is not only modulated with leaf aperture but also on dose rate and gantry speed; 2400 MU/minute maximum dose rate gives the most aggressive time estimation. Ten minutes extra delivery time was added per extra arc for plan #4 due to the fact that the patient localization will need to be verified again and the gating window reset. The 30 minutes setup time and 10 minutes extra verification time were purely assumptions based on our first experience with a heart treatment. We simply doubled our regular setup time due to the involvement of the heart movement. This estimate is generous because we are assuming cardiac motion management may be more complex, and that with routine clinical implementation, the time is likely to become less.

**Table 1:** System Descriptions and Parameters of the Four Plans.

Plan #	Treatment Platform	Collimator	Plan Technique	Beam Energy(MeV)	Beam Type	Dose Rate (MU/Minute)	Tracking Method
1	CyberKnife® G4™	IRIS^TM^	Non-isocentric plan	6	Photon FFF	1000	Synchrony® tracking
2	CyberKnife® M6™	InCise 2^TM^	Non-isocentric plan	6	Photon FFF	1000	Synchrony® tracking
3	TrueBeam^TM^ STx	HD 120^TM ^MLC	1 Arc VMAT	10	Photon FFF	2400	Gating
4	TrueBeam^TM^ STx	HD 120^TM ^MLC	3 Arc non-coplannar VMAT	10	Photon FFF	2400	Gating

## Results

**Figure 2:**
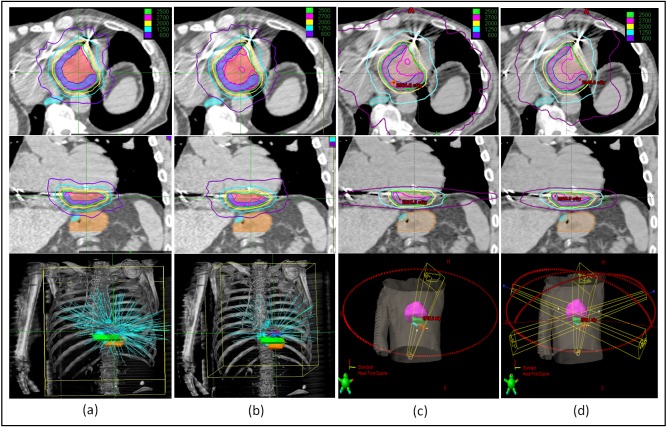
Isodose Distribution and Beam Arrangement for the Four Plans (a) CyberKnife® G4 plan with Iris collimator, (b) CyberKnife® M6 plan with InCise 2^TM^ MLC, (c) TrueBeam^TM^ STx VMAT plan using one full axial arc, (d) TrueBeam^TM^ STx VMAT plan with one axial full arc and two anterior partial arcs (120 degree) with a 10-degree couch kick on each side.

**Figure 3:**
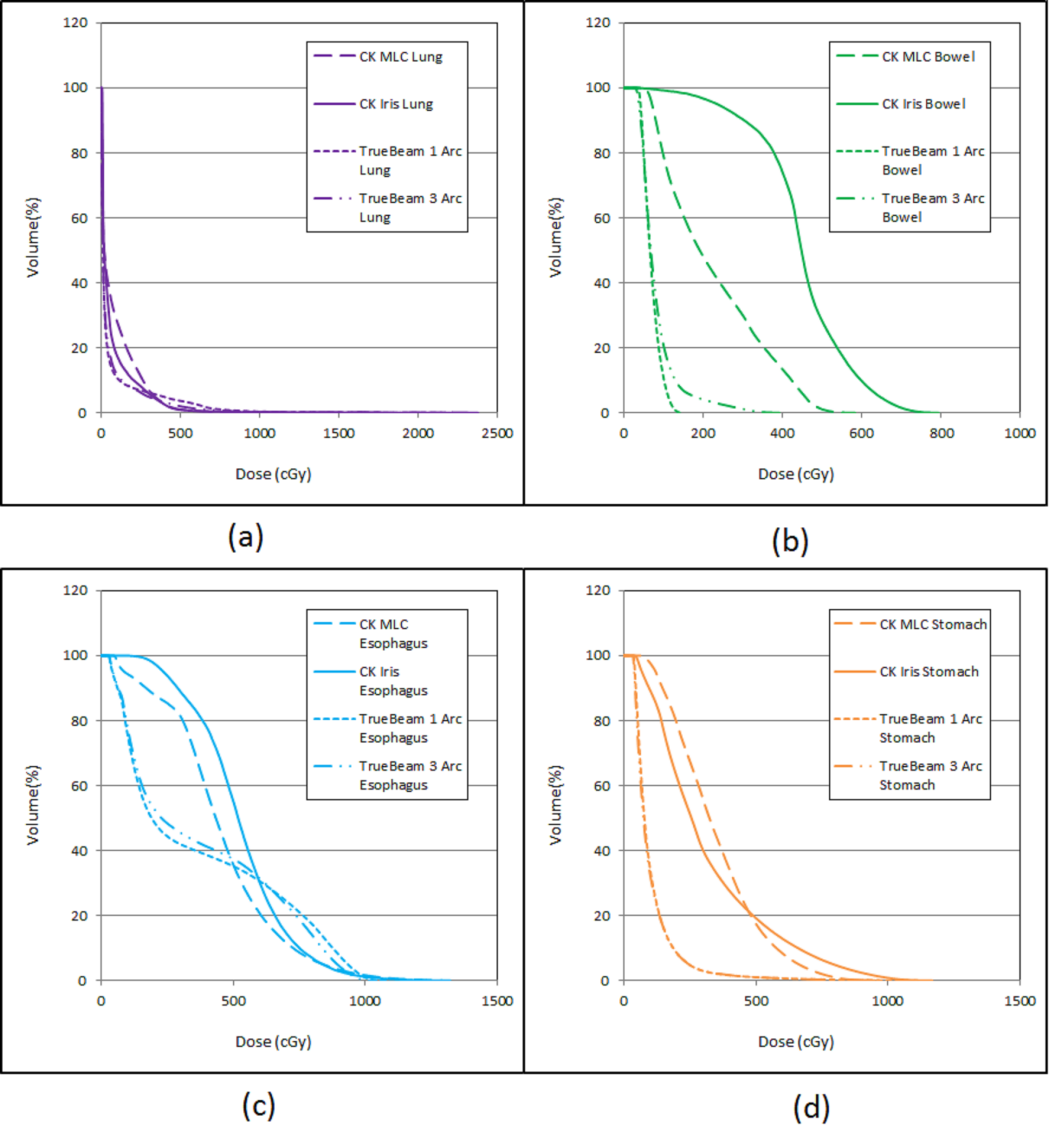
DVH Comparison of the Nearby Critical Structures of the Four Plans. (a) total lung, (b) bowel, (c) esophagus, (d) stomach.

We were able to create clinically acceptable plans on all three delivery platforms. The isodose distributions and the beam arrangement were compared (Figure 2), with plan statistics in Table 2. The four plans (Plans #1-#4) delivered 25 Gy to PTV with isodose lines of 75.0%, 83.0%, 84.3%, and 84.9% respectively, with conformity indices of 1.19, 1.16, 1.05, and 1.05 respectively. Plans with MLC have better uniformity in dose distribution in general. The dose gradients were 3.62, 3.42, 3.93, and 3.73 for plans #1-#4, respectively. The CyberKnife® MLC plan (Plan #2) had the best dose gradient, and the VMAT 1 Arc plan (Plan #3) had the worst. However, the difference was not significant, and the results were based on one patient only. The dose to nearby critical structures (lung, stomach, bowel, and esophagus) were within tolerance with slight differences due to the system beam arrangement, with their DVHs compared in Figure 3. The MUs of the four plans were 27671, 16522, 6275, and 6004, respectively; and the estimated total treatment times/beam delivery times were 99/69, 65/35, 37/7, and 56/6 minutes, respectively. The CyberKnife® plan with InCise 2^TM^ collimator had reduced MU (40% reduction) and beam delivery times (49% reduction) compared to the respective MU and beam delivery times using an Iris plan. VMAT plans were more efficient in MU usage (22% of the CyberKnife® Iris plan and 35% of the CyberKnife® MLC plan) and delivery time.

**Table 2:** Plan Statistics for Plans #1-#4 The delivery times in parentheses are the beam delivery times excluding setup time. The Target Composite is the ITV with a 5 mm margin to compensate for heart movement.

Plan #	System Description	Rx isodose line(%)	PTV Coverage(%)	Target Composite Coverage(%)	CI	Dose Gradient	MU	Delivery Time(Minutes)
1	CyberKnife® Iris	75	96.8	95.3	1.19	3.63	27671	99(69)
2	CyberKnife® MLC	83	97.7	96.9	1.16	3.42	16522	65(35)
3	TrueBeam STx^TM^ 1 Arc	84.3	97	98.4	1.05	3.93	6275	37(7)
4	TrueBeam STx^TM^ 3 Arc	84.9	97	98.8	1.05	3.73	6004	56(6)

## Discussion

Clinically acceptable plans can be created with all three platforms. While plans are all conformal, we found that VMAT plans can follow the target shape (lower conformity index) better than CyberKnife® plans with significantly fewer MUs. This is due to the fact that VMAT plans deploy direct aperture optimization and utilize higher penetrating radiation. The Iris plan is a cone-based plan, which on average has the least efficiency in MU delivery due to the absence of irregular beam shapes. With the InCise 2^TM^ MLC, irregular fields are now available which help to increase the delivery efficiency [17-19]; however, the current optimizer does not directly optimize the beam aperture, which limits the delivery efficiency on Plan #2. When MLC is applied, the beam set is pre-generated based on the shape of the target (perimeter shapes, eroded shapes, and random shapes) and are treated as fixed aperture during plan optimization in the CyberKnife® MultiPlan. In the Eclipse planning system, the VMAT plan is directly optimized on beam apertures and weights, which produce better conformity and MU efficiency. In this study, VMAT plans using 10 MV photons yield approximately a 15% reduction in MUs as opposed to 6 MV (the only option for CyberKnife® system). Percent depth dose at 10 cm depth is 61% and 71% for CyberKnife 6 MV beam with a 60 mm cone and TrueBeam 10MV FFF beam in a 10 cm by 10 cm field.

Another difference between the CyberKnife® system and the TrueBeam system is the treatment space. One of the advantages of the CyberKnife®  system is that there is no isocenter. The compact X-band linear accelerator can move around the patient freely with the six-joint robotic arm. It can access more oblique angles from superior and inferior directions although lacks posterior beams. The Gantry-based TrueBeam^TM^ STx system is better in axial isocentric arc delivery. The introduction of a non-coplanar partial arc in Plan #4 provided some superior and inferior oblique beams, but it significantly complicated the treatment. Due to the beam arrangement differences between the systems, isodose distributions for the CyberKnife® plans are observed to have a dose drop-off more uniformly over all the directions, which yield a better dose gradient, and the TrueBeam VMAT plans have a dose distribution stretch-out in the axial plane. As a rough quantitative measure of this stretch-out effect, the average diameters (averaged in lateral and anterior to posterior directions) of the 12.5 Gy line were measured on the central axial plane. They were 8.9 cm, 8.8 cm, 10.5 cm, and 10.0 cm for plans #1-#4, respectively. The three arc plan (Plan#4) had less axial dose stretch-out, but still could not compete with the CyberKnife® plans. The CyberKnife® MLC plan (Plan #2) had a better dose drop off with fewer MUs and delivery time than the CyberKnfe Iris plan (Plan #1), which is consistent with other studies [17-19].

Other than the differences discussed above, the major difference between the CyberKnife® and the TrueBeam^TM^ STx plans is the patient localization and motion tracking during the delivery. During pre-treatment assessment for this patient, we found the ICD tip was not trackable with the CyberKnife®. Therefore, a temporary pacing wire (Oscor, Inc., Miami Lakes, FL) was placed in the RV apex as an imaging fiducial marker. A fixed-helix unipolar lead design was used to minimize imaging artifact and was placed using a sterile percutaneous approach via the right axillary vein into the RV apex under fluoroscopic guidance. This location was chosen to avoid both tracking interference from the patient’s existing ICD lead and to place the fiducial marker as close to the target tissue as possible. The fiducial marker was removed after STAR under fluoroscopic guidance. The Synchrony tracking primarily compensates the heart motion caused by the respiratory movement. Tracking accuracy was later analyzed using the system log file and was found to be close to our estimation [21-22]. The motion management on the TrueBeam^TM^ STx, on the other hand, does not have strict requirements on markers as does the CyberKnife® system. The TrueBeam^TM^ STx system utilizes kV fluoroscopic imaging and CBCT for target localization. In this case, the existing ICD lead could have served as the fiducial marker for fluoroscopic image guidance so that the invasive procedure to place the extra lead would not have been needed; however, benefits may possibly be moderated by a worse intermediate dose conformity index and more operator-dependent motion management by gated delivery [23-24]. Motion management is not as automated as with the CyberKnife® and is, therefore, more operator dependent.

## Conclusions

We compared STAR treatment plans between three treatment platforms. All plans were clinically acceptable regarding target coverage and critical structure sparing. Plans with MLC were considerably more efficient in MUs and delivery time. The recently released InCise 2^TM^ collimator with the M6 system provided the most conformal plan (steepest dose drop-off) with significantly reduced MUs and treatment time. VMAT plans were most efficient in MUs and delivery time. Fluoroscopic image guidance removes the need for additional fiducial marker placement; however, benefits may possibly be moderated by a worse intermediate dose conformity index and more operator-dependent motion management by gated delivery.
